# Supplementary material for: Large model structural uncertainty in global projections of urban heat waves
Source: Nat Commun. 2021 Jun 18;12:3736. doi: 10.1038/s41467-021-24113-9 (PMC8213750; doi:10.1038/s41467-021-24113-9)
Supplement: Supplementary file 1 — Supplementary Information for "Large model structural uncertainty in global projections of urban heat waves" [file 41467_2021_24113_MOESM1_ESM.pdf]

Supplementary Information

for

Large model structural uncertainty in global projections of urban heat waves

Zhonghua Zheng[1], Lei Zhao *[1,2], and Keith W. Oleson[3]

[1]Department of Civil and Environmental Engineering, University of Illinois at Urbana-Champaign, Urbana, IL 61801, USA.

[2]National Center for Supercomputing Applications, University of Illinois at Urbana-Champaign, Urbana, IL 61801, USA.

[3]Climate and Global Dynamics Laboratory, National Center for Atmospheric Research, Boulder, CO 80305, USA.

*Correspondence to: leizhao@illinois.edu

Supplementary:

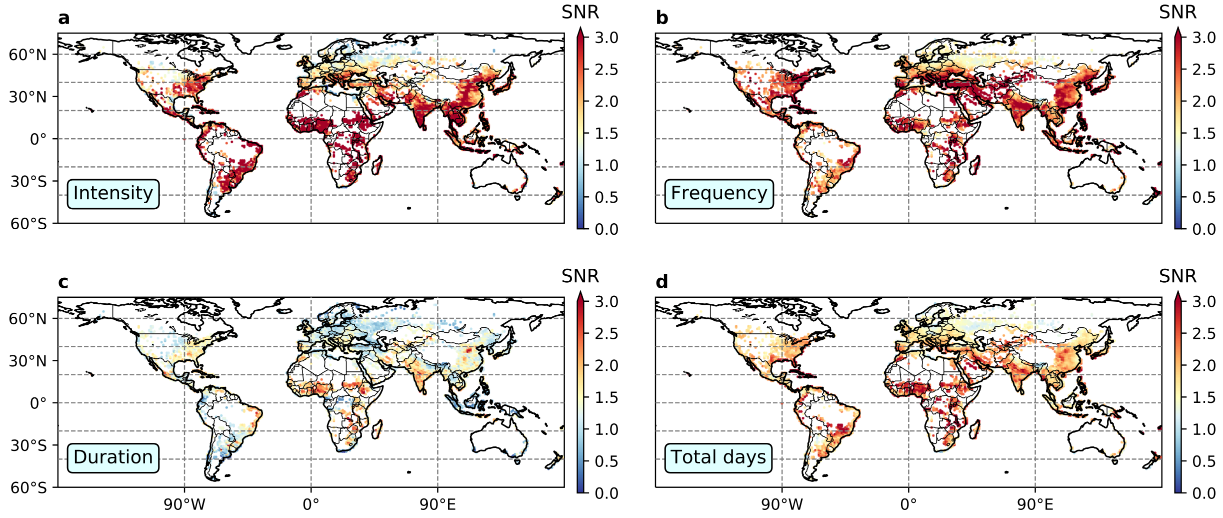

Supplementary Figure 1: **Signal-to-noise ratio (SNR) of the mean change in urban heat waves (a) intensity, (b) frequency, (c) duration, and (d) total days in 2061–2070 relative to 2006–2015.**

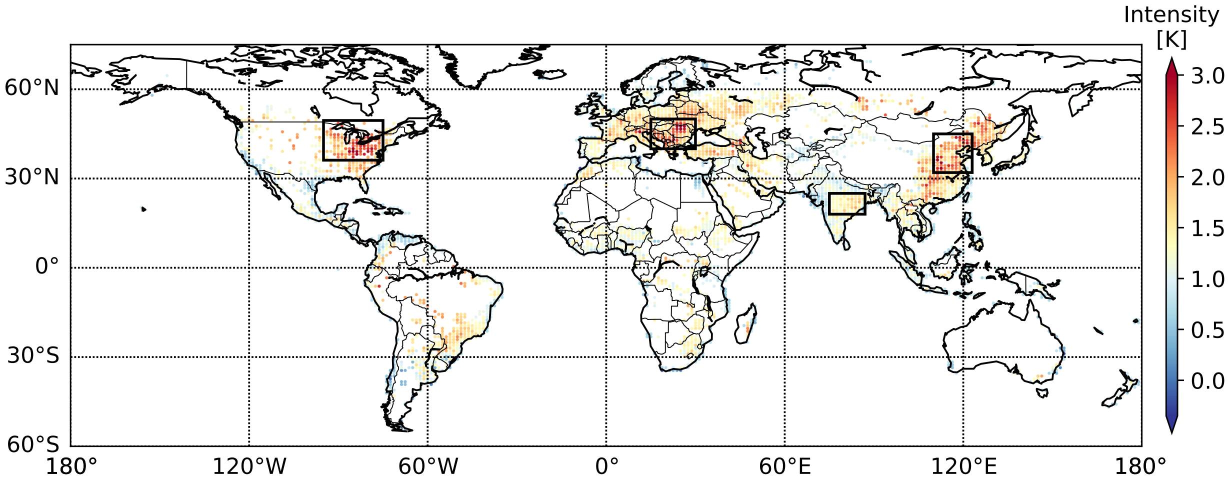

Supplementary Figure 2: **Four "hotspot" regions identified based on the projection of substantial increase in urban heat waves intensity and frequency with high inter-model confidence.** Each colored point represents multi-model mean change in average urban heat waves intensity, same with Fig. 2a. Rectangular boxes outline the four hotspot regions: Great Lakes region (36.15–49.5°N, 95–75°W), Southern Europe (40–50°N, 15–30°E), Central India (18–25°N, 75–87°E), and North China (32–45°N, 110–123°E).

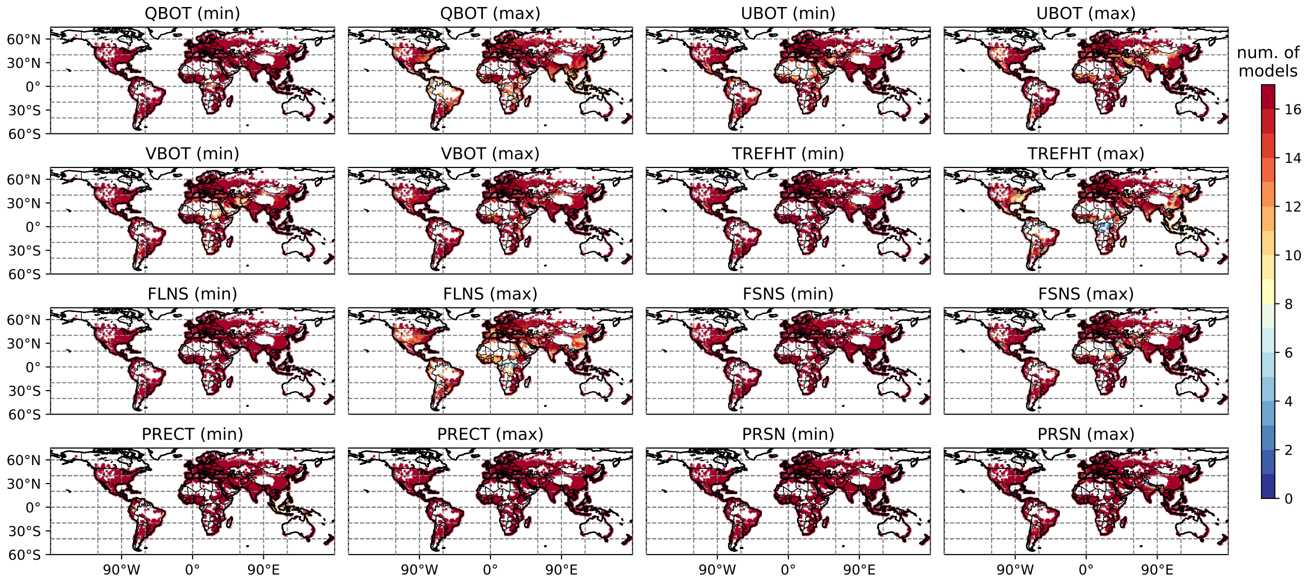

Supplementary Figure 3: **The range of forcing variables in CMIP5 models well covered by CESM training data (10% of the 2061–2070 data from each member).** The colors indicate the number of CMIP5 models out of 17 in total with the ranges of the forcings (2.5th percentile and 97.5th percentile) covered by the original CESM training data. QBOT: near-surface specific humidity, kg/kg; UBOT: Eastward near-surface wind, m/s; VBOT: Northward near-surface wind, m/s; TREFHT: near-surface air temperature, K; FLNS: surface net longwave radiation, W/m^2; FSNS: surface net shortwave radiation, W/m^2; PRECT: precipitation, m/s; PRSN: snowfall flux, m/s.

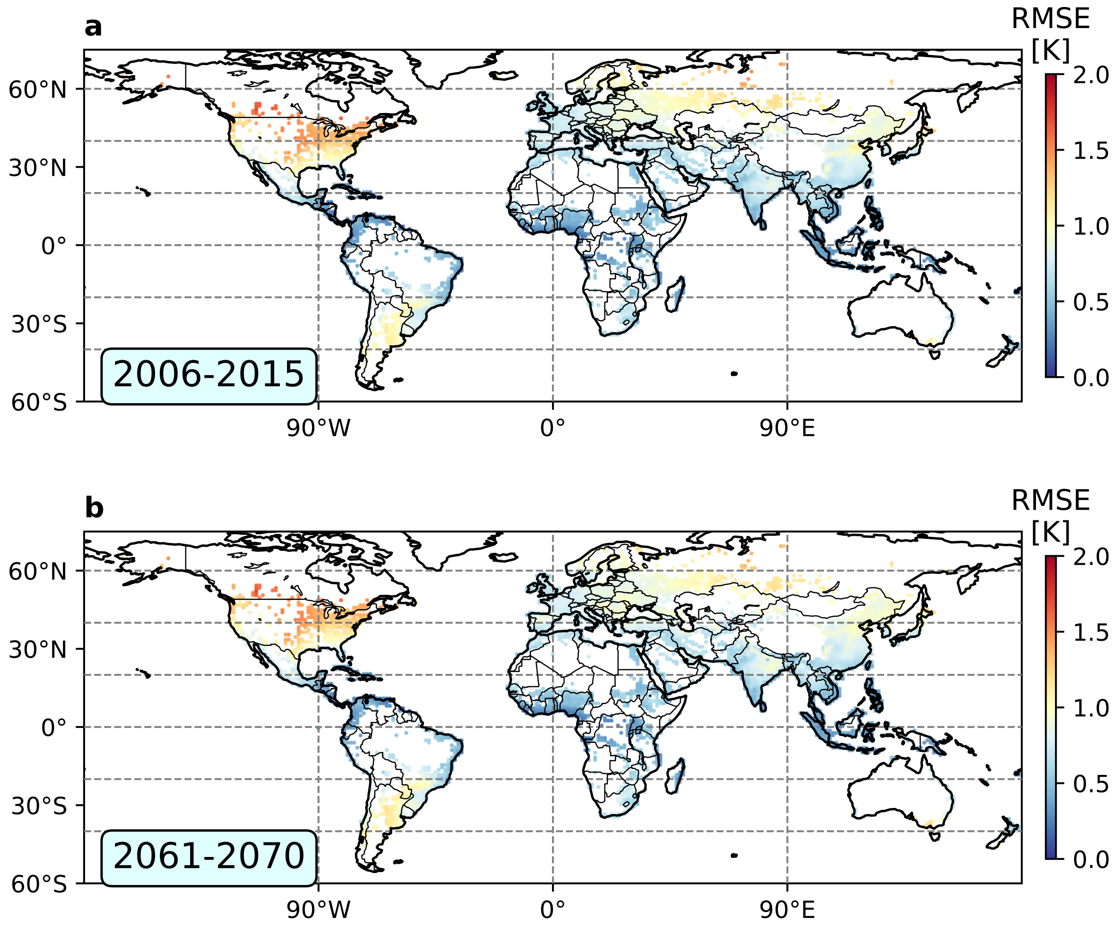

Supplementary Figure 4: **Average root-mean-square-error (RMSE) in temperature validation of the emulator across 32 CESM ensemble member runs (member #2–#33).** The "error" in RMSE denotes the difference (K) between daily temperatures dynamically modeled by the CESM ensemble members and the ones modeled by the emulator. The average RMSE was calculated based on the 32 CESM ensemble member runs from (a) 2006–2015, and (b) 2061–2070.

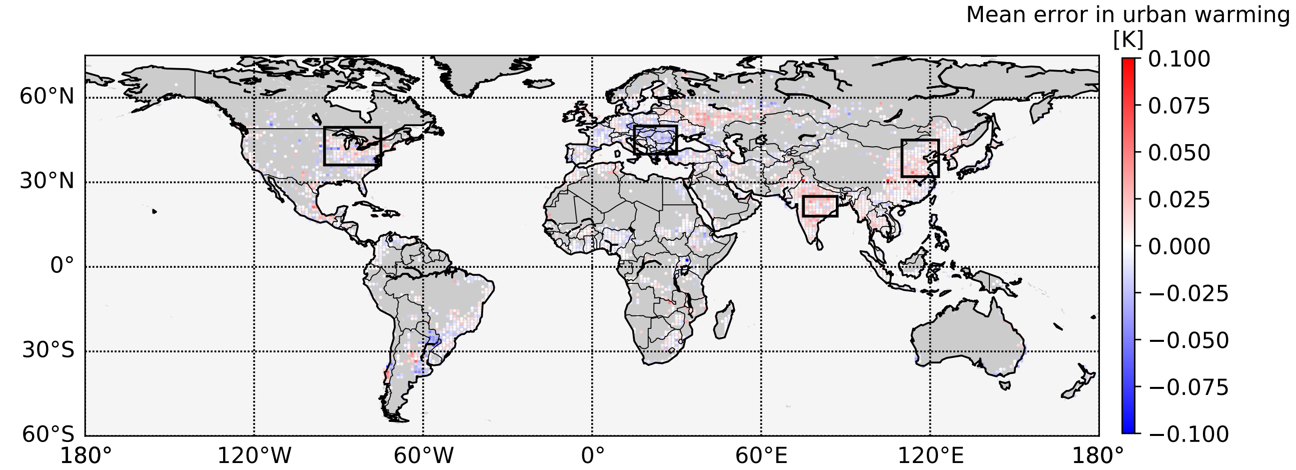

Supplementary Figure 5: **Mean error in emulating the CESM modeled multi-member mean urban warming in daily maximum temperature (2061–2070 relative to 2006–2015).** "Error" here is defined as the difference (K) between the emulated and the CESM-LE modeled multi-member mean urban daily maximum temperature. Four boxes are same with Supplementary Fig. 2, indicating the four hotspot regions: Great Lakes region (36.15–49.5°N, 95–75°W), Southern Europe (40–50°N, 15–30°E), Central India (18–25°N, 75–87°E), and North China (32–45°N, 110–123°E).

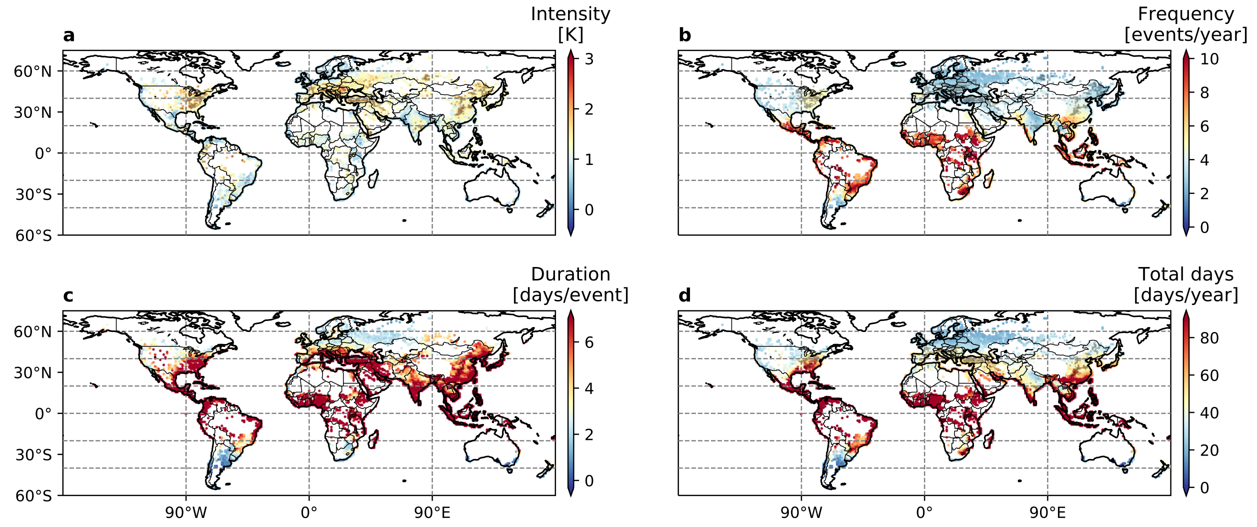

Supplementary Figure 6: **Multi-model ensemble mean change in average urban heat wave (based on minimum temperature) (a) intensity (K), (b) frequency (events per year), (c) duration (days per event), and (d) total days (days per year) in 2061–2070 relative to 2006–2015 (17 selected Earth system models and the first member of the CESM-LE runs).** Stippling indicates substantial change (intensity > 1.5 K) with high inter-model robustness (SNR > 2.0).

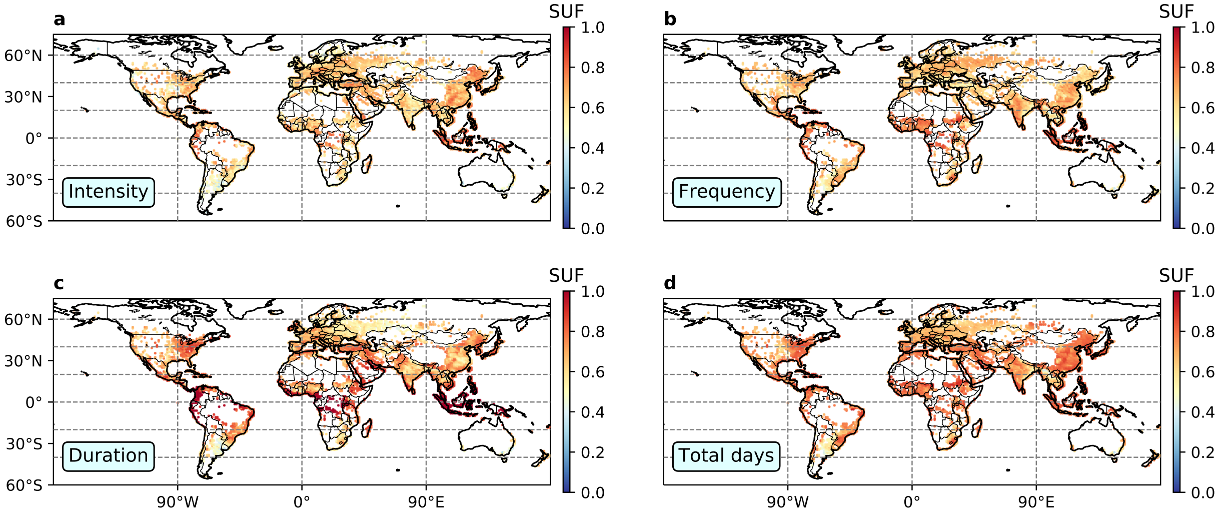

Supplementary Figure 7: **Relative contribution of the model structural variability in urban heat wave projections (based on minimum temperature) in 2061–2070 relative to 2006–2015 under RCP 8.5.** (a) intensity; (b) frequency; (c) duration; and (d) total days. Each colored point represents a decadal mean structural uncertainty fraction (SUF) defined as $\frac{\sigma_{\mathrm{CMIP}}}{\sigma_{\mathrm{CMIP}}+\sigma_{\mathrm{CESM}}}$ within a 0.9° (lat.) $\times$ 1.25° (lon.) model grid cell. σ_{CMIP} denotes the standard deviation across multi-model projections and σ_{CESM} denotes the standard deviation across multi-member projections.

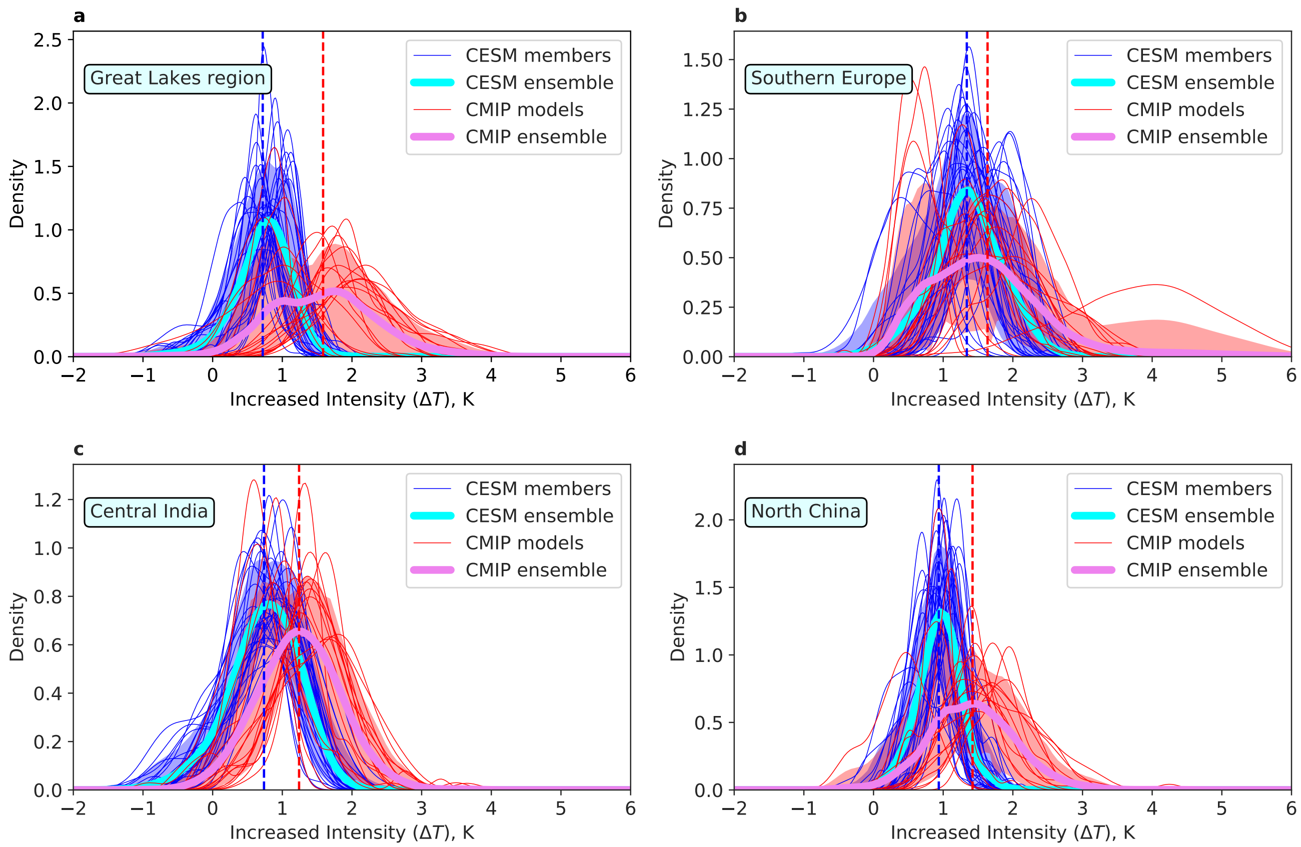

Supplementary Figure 8: **Probability distribution of changes in urban heat wave (based on minimum temperature) intensity by 2061–2070. The thick lines mark the CMIP5 multi-model mean (thick violet lines) and CESM-LE multi-member mean (thick cyan lines).** The thin red lines mark individual models of CMIP5; the thin blue lines mark individual CESM-LE members. The red and blue shading denote the 5th to 95th percentile across CMIP5 models and across CESM members respectively for each small bin. The changes are derived from land grid points in (a) Great Lakes region (36.15–49.5°N, 95–75°W), (b) Southern Europe (40–50°N, 15–30°E), (c) Central India (18–25°N, 75–87°E), and (d) North China (32–45°N, 110–123°E) between the 2006–2015 and 2061–2070.

Supplementary Table 1: List of CMIP5 ESMs analyzed in this study.

**Model ID**	**Model Name**	**Original Resolution (lat. $\times$ lon.)**
1	ACCESS1-0	1.25×1.875
2	ACCESS1-3	1.25×1.875
3	CanESM2	2.7906×2.8125
4	CNRM-CM5	1.4008×1.40625
5	CSIRO-Mk3-6-0	1.8653×1.875
6	FGOALS-s2	1.6590×2.8125
7	GFDL-CM3	2×2.5
8	GFDL-ESM2G	2.0225×2
9	GFDL-ESM2M	2.0225×2.5
10	HadGEM2-CC	1.25×1.875
11	HadGEM2-ES	1.25×1.875
12	IPSL-CM5A-MR	1.2676×2.5
13	MIROC5	1.4008×1.40625
14	MIROC-ESM	2.7906×2.8125
15	MIROC-ESM-CHEM	2.7906×2.8125
16	MRI-CGCM3	1.12148×1.125
17	MRI-ESM1	1.12148×1.125
18	CESM	0.9424×1.25
